# NEK8 regulates colorectal cancer progression via phosphorylating MYC

**DOI:** 10.1186/s12964-023-01215-z

**Published:** 2023-08-18

**Authors:** Beibei Cao, Kailun Zhang, Changjie Pan, Yifei Dong, Feng Lu

**Affiliations:** 1 Department of Breast Surgery, Henan Provincial People’s Hospital, Zhengzhou City, China; 2 Zhengzhou University People’s Hospital, Zhengzhou City, China

**Keywords:** NEK8, MYC, Colorectal cancer

## Abstract

Radiotherapy and chemotherapy remain the mainstay of treatment for colorectal cancer (CRC), although their efficacy is limited. A detailed understanding of the molecular mechanisms underlying CRC progression could lead to the development of new therapeutic strategies. Although it has been established that MYC signaling is dysregulated in various human cancers, direct targeting MYC remains challenging due to its "undruggable" protein structure. Post-translational modification of proteins can affect their stability, activation, and subcellular localization. Hence, targeting the post-translational modification of MYC represents a promising approach to disrupting MYC signaling. Herein, we revealed that NEK8 positively regulates CRC progression by phosphorylating c-MYC protein at serine 405, which exhibited enhanced stability via polyubiquitination. Our findings shed light on the role of NEK8/MYC signaling in CRC progression, offering a novel and helpful target for colorectal cancer treatment.

Video Abstract

**Supplementary Information:**

The online version contains supplementary material available at 10.1186/s12964-023-01215-z

## Introduction

In recent years, the incidence of colorectal cancer (CRC) has significantly increased, becoming the third most common tumor and the fourth leading cause of cancer-related death worldwide [1, 2]. Surgery, perioperative radiotherapy, and chemotherapy are the main therapeutic methods for colorectal cancer patients [1, 3]. However, the therapeutic effect of radiotherapy and chemotherapy is often poor for advanced CRC, emphasizing the importance of understanding the underlying pathogenesis of CRC for identifying novel and effective therapeutic strategies.

It has been established that Never-in-mitosis A-related kinase 8 (NEK8), also called NPHP9 or NEK12A, belongs to the serine/threonine protein kinase family [4]. Previous studies have substantiated that NKE8 plays a critical role in living cells, regulating the cell cycle, primary cilia disassembly, and DNA damage response [5–7]. Additionally, NEK8 reportedly has varying effects on the progression of different diseases and has been shown to positively enhance the proliferation and migration capabilities of gastric cancer cells. Moreover, NEK8 overexpression has been linked to obesity associated with type 2 diabetes (T2DM) by stimulating adipocyte proliferation. Mutations, dysfunction, or abnormal expression of NEK8 have been associated with severe syndromic renal cystic dysplasia and neonatal cholestasis. Furthermore, NEK8 has been found to impact the progression of breast cancer and glioma [8–13]. However, no research has assessed the biological role of NEK8 in colorectal cancer. Therefore, it is important to investigate whether NEK8 plays a role in the development and progression of colorectal cancer.

c-MYC and its homologous proteins (N-MYC and L-MYC) belong to the MYC oncogene family [14, 15]. More than 50% of human cancers have MYC signaling dysfunction, which is reportedly correlated with poor overall survival in cancer patients [14, 16, 17]. It is now understood that there are three critical domains in MYC protein: the amino-terminal domain (NTD), which is essential for the transcriptional activation of MYC protein; the basic helix-loop-helix (HLH) domain, which is for DNA binding; and the carboxy-terminal domain (CTD) [18]. MYC signaling participates in a broad spectrum of intracellular biological processes of cancer cells, including proliferation, migration, invasion, differentiation, apoptosis, DNA damage response, and metabolism, which contributes to the development of different kinds of cancer [15, 17, 18]. Despite the challenges in directly targeting MYC, researchers have explored alternative methods to disrupt MYC protein function and expression. This includes strategies such as inhibiting MYC/Max complex formation, suppressing MYC transcription and/or translation, and destabilizing MYC protein. Post-translational modifications are also an important mechanism that can regulate MYC protein stabilization. For instance, proteins such as Trim6, FBXW7, and USP28 can regulate MYC poly-ubiquitination, while PIM2, CDK2, DYRK2, GSK3, and PPP2CA can affect the phosphorylation status of MYC, thereby impacting its stability [19–27]. Despite the potential of inhibitors targeting the post-translational modification factors of MYC protein, their clinical application is limited due to side effects or poor efficacy. Hence, a deeper understanding of the post-translational modification of MYC might provide novel and effective therapeutic strategies for cancer patients.

Herein, we substantiated that NEK8 was upregulated in colorectal cancer compared with normal colorectal tissues. Then, in vivo and in vitro experiments demonstrated that NEK8 expression was positively correlated with colorectal cancer proliferation, and loss of NKE8 inhibited the growth of the subcutaneous transplanted tumors in the nude mice. Further, we revealed that NEK8 could activate MYC signaling by restricting the poly-ubiquitination of MYC. Moreover, we found that NEK8 mediated MYC stabilization by phosphorylating MYC at serine 405 phosphorylation. Besides, we uncovered that NEK8-induced cancer cell proliferation and colorectal cancer progression depend on the serine 405 phosphorylation of MYC by in vivo or in vitro assays. Collectively, these findings imply that the NEK8/MYC signaling pathway might be a novel target for colorectal cancer treatment.

## Material and methods

### Agents and cell lines

SW48, Lovo, and HEK 293 T cell lines were purchased from American Type Culture Collection (ATCC). SW48, Lovo, and HEK 293 T cell lines were cultured with dulbecco's modified eagle medium (DMEM) plus 10% (v/v) foetal bovine serum (FBS) and 1% (v/v) penicillin–streptomycin solution in 5% CO2 air chamber at 37 °C. Antibodies for NKE8 (ab153879, Abcam), GAPDH (A19056, ABclonal), HA-tag (3724, Cell Signaling Technology), c-MYC (A1309, ABclonal), Cyclin D1 (55,506, Cell Signaling Technology), CDK4 (12,790, Cell Signaling Technology), and His-tag (12,698, Cell Signaling Technology) were obtained from commercial companies. Antibodies for P–c-MYC S405 were produced by ABclonal company. Anti-Flag Magnetic Beads (HY-K0207) and Protein A/G Magnetic Beads (HY-K0202) were purchased from MedChemExpress company.

### Western blot assays

Pre-treated cells were lysed on ice using NP-40 lysis buffer (P0013F, Beyotime) for 10 min. Then, the cell lysate was centrifugated for 10 min at 14,000 g. Protein concentration was measured using a bicinchoninic acid assay kit (Thermo). The protein was separated using SDS-PAGE electrophoresis and then transferred to PVDF membranes. Membranes were cut into bands according to the molecular weight of identified proteins. Bands were cultured with primary antibodies at 4 °C overnight. Then, the bands were washed using TBST buffer three times. Next, bands were incubated with HRP- conjugated secondary antibodies at room temperature for 2 h. The protein signaling was detected using Enhanced chemiluminescence (ECL). Then, the images were collected and analyzed.

### Immunoprecipitation assay

c-MYC antibody was incubated with Protein A/G Magnetic Beads at room temperature for 2 h. After washing the magnetic beads three times, cellular lysates were added to the magnetic beads. The mixture was then rotated overnight at 4 °C for protein binding. Finally, western blot assays were conducted.

### Real-time PCR

Total RNAs were extracted using RNA Trizol Reagent (15,596,026, ThermoFisher). RNAs were reversed into cDNAs using the ABScript II cDNA First-Strand Synthesis Kit (RK20400, ABclonal). Then, Genious 2X SYBR Green Fast qPCR Mix (RK21204, ABclonal) was used for quantitive real-time PCR. The primers were listed as followed: NKE8 forward: 5-CTTCGTGCAGATCCTGCTTG-3, Reverse: 5-GGAGATGCCGAAATCACCGAT-3; c-MYC forward: 5-GGCTCCTGGCAAAAGGTCA-3, Reverse: 5- CTGCGTAGTTGTGCTGATGT-3.

### Immunohistochemistry (IHC)

Colorectal cancer samples were obtained from Henan Provincial People's Hospital. This study was approved by Medical Ethics Committee of Henan Provincial People's Hospital (2,021,182). Informed consent was obtained from CRC patients before the study. The immunohistochemistry assays were performed in collaboration with Biossci Biotechnology Company, which provided assistance and expertise in conducting the experiments. The results of IHC were assessed by two experienced pathologists independently. The proportion score was calculated according to the fraction of positively stained tumor cells: 1 (< 10%); 2 (10%-50%; 3(50–75%); 4 (> 75%), and the intensity reflected the staining intensity: 0, no staining; 1, weak; 2, intermediate; 3, strong. The IHC score was quantified by multiplying the proportion score with the intensity score.

### CCK8 assays

CCK8 assays were conducted using Cell Counting Kit-8 (RM02823, ABclonal) according to the manufacturer's instructions.

### EdU assays

The proliferation ability of identified cancer cells was assessed using BeyoClick™ EdU Cell Proliferation Kit with DAB (Beyotime) according to the manufacturer's instructions.

### Quantification of adenosine 5'-triphosphate (ATP)

Cellular ATP content was measured using the ATP Content Assay Kit (AKOP004, Boxbio) according to the manufacturer's instructions.

### Cell lines construction

To construct gene overexpression cell lines, a three-package system consisting of pLVX-genes, MD2-G, and PPAX plasmids was used for overexpression lentivirus. A three-package system comprising pLKO.1-shRNAs, MD2-G, and PPAX plasmids was applied for silencing lentivirus generation. The following sequence was the target of c-MYC: shc-MYC#1 (5- CCATAATGTAAACTGCCTCAA-3); shc-MYC#2 (5- CAGTTGAAACACAAACTTGAA-3); shc-MYC#2 (5- CAGGAACTATGACCTCGACTA-3). The pSpCas9(BB)-2A-Puro (PX459) V2.0 plasmid was used to construct gene knockout cell lines. The following sequence was the target of NEK8: sgRNA#1 (5- GATGACCAAGGAAGAGCGGC-3); sgRNA#2 (5- GGTCAGCCTTTCGCAGGCAC-3); sgRNA#3 (5- GGTGCACAATCCTAGGGATA-3). Puromycin (2 ug/ml) was used to screen the infected cells. Western blots or RT-PCR assays were conducted to verify the successful construction of cell lines.

### Animal assays

The mouse xenograft assays were approved by Animal ethics committee of Henan Provincial People's Hospital (2,020,098). 4-week-old male nude mice were obtained from Beijing Huafukang Bioscience Company. Pre-treated SW48 cancer cells were subcutaneously injected into the back of nude mice. Tumor volume was measured at the indicated times. Tumor volume was calculated using 0.5 × Length (L) × Width (W) × Width (W). After the experiment, tumors were isolated, weighed, and fixed with 4% paraformaldehyde, followed by western blot assays or immunohistochemistry.

### Statistical analysis

Data were retrieved from online databases, including The Cancer Genome Atlas (TCGA; http://xena.ucsc.edu/welcome-to-ucsc-xena/), GEPIA (http://gepia.cancer-pku.cn/index.html), and Kaplan–Meier Plotter (http://kmplot.com/analysis/). Datasets GSE17537, GSE24551, and GSE29623 underwent GSEA analysis using GSEA software (http://www.gsea-msigdb.org/gsea/index.jsp). GENEMANIA (http://genemania.org/) database was used to detect the potential binding of NEK8 and c-MYC. All data were analyzed using SPSS 20.0 software and GraphPad Prism 9.0 software. Differences were analyzed using the Student's t-test for two groups or ANOVA for multiple groups. The correlation between variables was assessed using either Chi-Square or Pearson's correlation test. A *P*-value < 0.05 was statistically significant.

## Results

### NEK8 was overexpressed in colorectal cancer

**Fig. 1:**
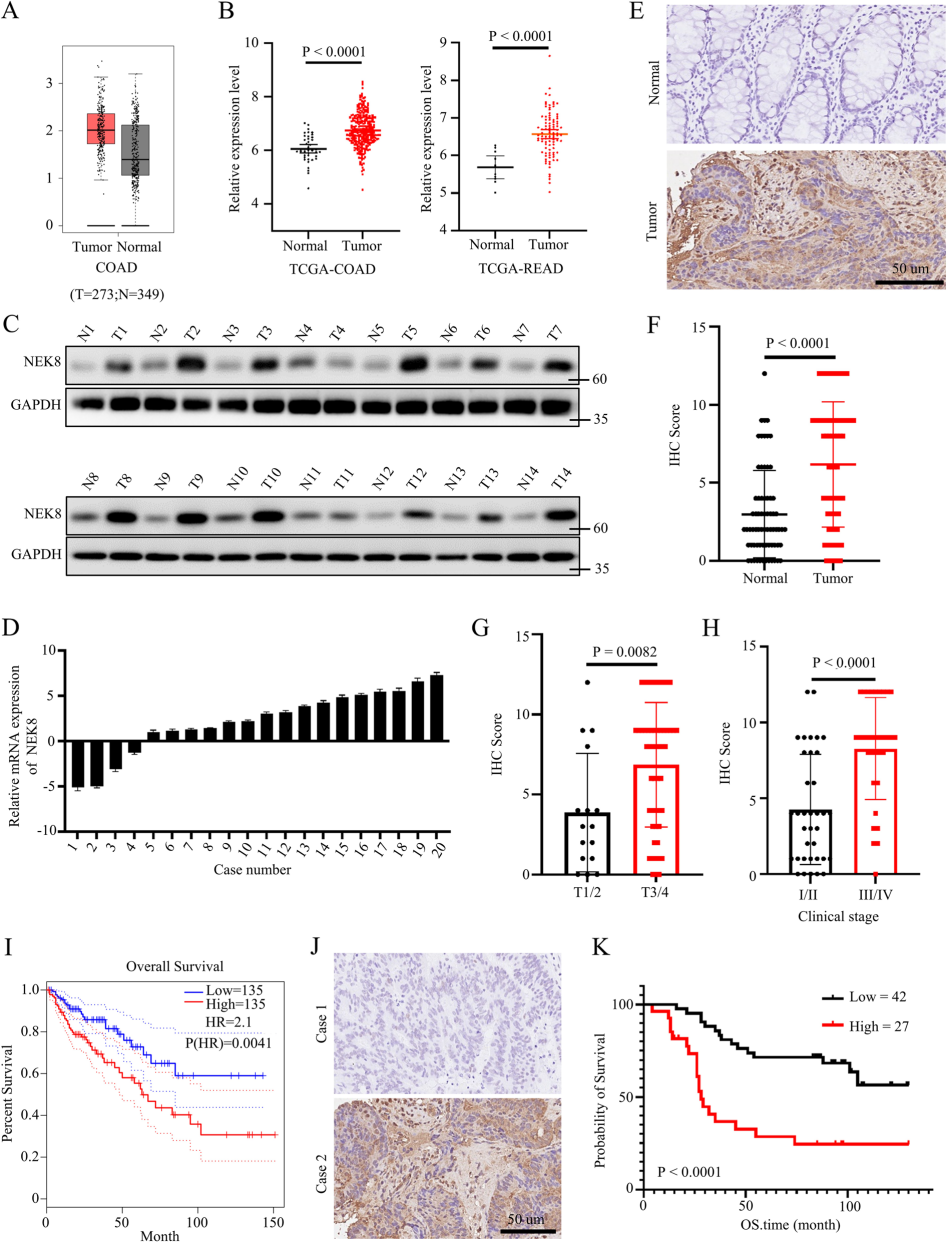
NEK8 was upregulated in colorectal cancer. **A** Assessing the expression level of NEK8 in colorectal cancer tissues and normal colorectal tissues using the GEPIA database. **B** Analysis of the expression level of NEK8 in colorectal cancer tissues and normal colorectal tissues using TCGA database; *P* < 0.0001. **C** Analysis of the expression level of NEK8 in colorectal cancer tissues and adjacent normal colorectal tissues by western blots. **D** Analysis of the mRNA expression level of NEK8 in colorectal cancer tissues and adjacent normal colorectal tissues by RT-PCR. **E**–**H** Analysis of the expression level of NEK8 in colorectal cancer tissues and adjacent normal colorectal tissues by IHC assays; representative images shown (**E**); statistical analysis of IHC scores grouped by normal and tumor (**F**), T stage (**G**), and clinical stage (**H**); number = 69, *P* (**F**) < 0.0001, P (**G**) = 0.0082, and P (H) < 0.0001. **I** Analysis of the overall survival time of colorectal cancer patients grouped by NEK8 expression level using the GEPIA database; *P* = 0.0041. **J** IHC assays to detect the expression level of NEK8 in colorectal cancer tissues; representative images are shown. **K** Kaplan–Meier Plotter of colorectal cancer patients grouped by IHC score from Fig. 1** J**; *P* < 0.0001. All western blots were conducted three times, and similar results were found; a Student's t-test was applied for statistical analysis

To assess the role of NEK8 in colorectal cancer progression, we first analyzed the expression level of NEK8 in colorectal cancer and normal colorectal tissues using the GEPIA and TCGA databases. We found a higher expression level of NEK8 in colorectal cancer compared with normal colorectal tissues (Fig. 1A, B). Meanwhile, NEK8 was upregulated in multiple cancers (Figure S1A). Next, we detected the expression level of NEK8 in 14 pairs of colorectal cancer and adjacent normal tissues using western blots. Results showed that NEK8 was upregulated in colorectal cancer tissues compared with adjacent normal tissues (Fig. 1C). Moreover, RT-PCR demonstrated that colorectal cancer tissues had higher mRNA expression of NEK8 than adjacent normal tissues (Fig. 1D). Further, IHC assays were conducted to investigate the expression level of NEK8 in 69 pairs of colorectal cancer and adjacent normal tissues. The representative images are shown in Fig. 1E. Statistical analysis showed that NEK8 expression in colorectal cancer tissues was higher than in adjacent normal tissues, and patients with higher T stage or clinical stage had higher expression of NEK8 (Fig. 1F-H). Next, we analyzed the relationship between overall survival and NEK8 expression among CRC patients using the GEPIA database. Results showed that patients with higher NEK8 expression had poorer overall survival (Fig. 1I). Besides, we found that higher NEK8 expression had poorer overall survival time in other kinds of tumors, such as glioblastoma, low-grade glioma, and uveal melanoma (Figure S1B). Consistently, we investigated the correlation between overall survival and NEK8 expression in 69 cases of colorectal cancer patients. The representative images are shown in Fig. 1J. It was found that NEK8 expression was negatively correlated with overall survival (Fig. 1K). Taken together, our findings revealed that NEK8 is upregulated in colorectal cancer and suggest its potential role as an oncogenic factor in driving the progression of colorectal cancer.

### Overexpression of NEK8 promoted colorectal cancer cell proliferation

**Fig. 2:**
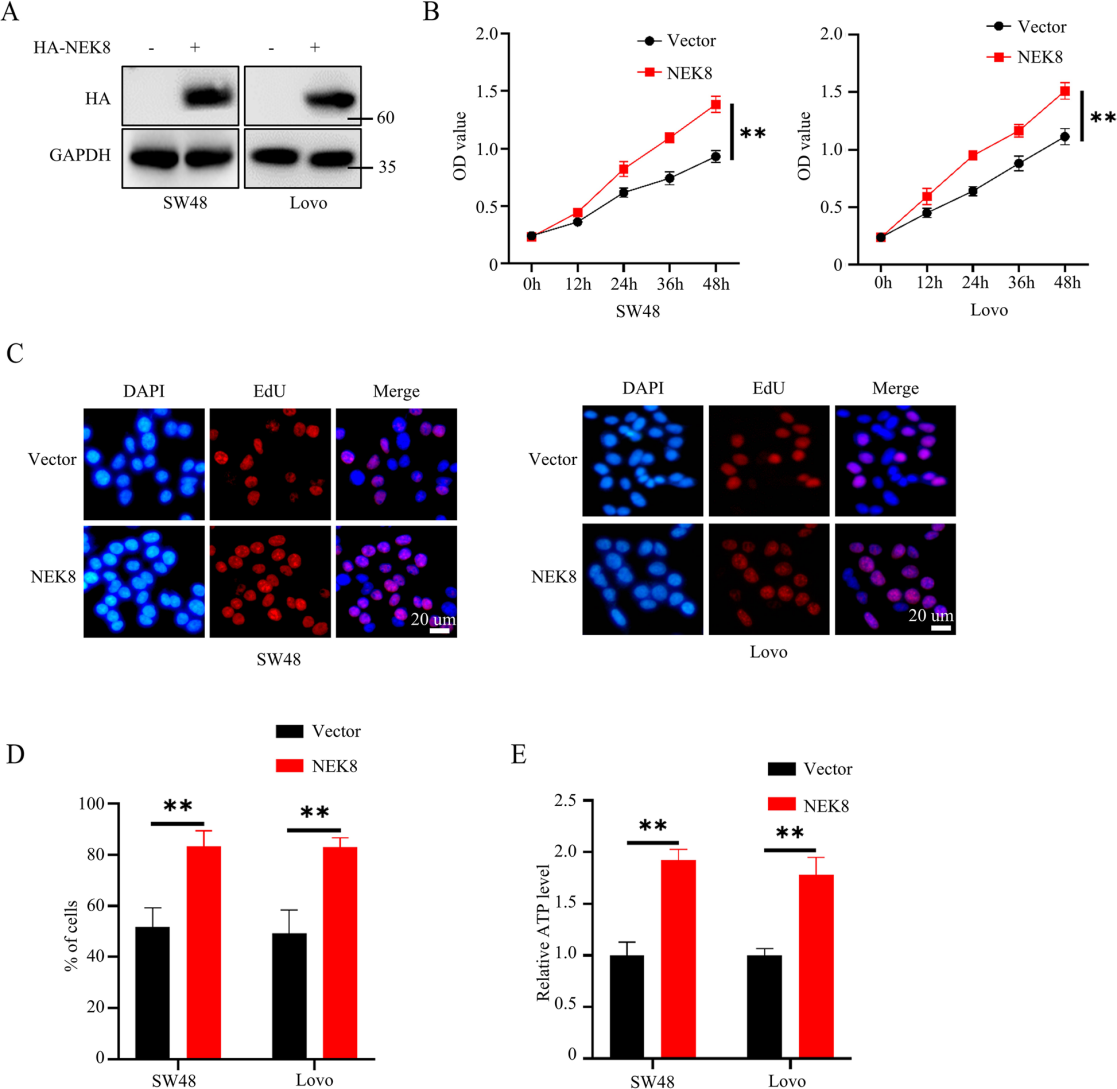
NEK8 overexpression promoted colorectal cancer cell proliferation. **A** Western blots confirmed the successful construction of SW48 and Lovo stably expressing Vector or NEK8. **B** CCK8 assays to investigate the proliferation ability of SW48 and Lovo stably expressing Vector or NEK8; *n* = 3, P(Left) = 0.0009, P(Right) = 0.0025. **C**, **D** EdU assays to investigate the DNA replication ability of SW48 and Lovo stably expressing Vector or NEK8; representative images shown (**C**); statistical analysis shown (**D**); *n* = 3, P(SW48) = 0.0047, P(Lovo) = 0.0039. **E** Detecting the cellular ATP level of SW48 and Lovo stably expressing Vector or NEK8; *n* = 3, P(SW48) = 0.0007, P(Lovo) = 0.0017. All western blots were conducted three times, and similar results were found; Student's t-test was applied for statistical analysis

To clarify the role of NEK8 in colorectal cancer, we first constructed SW48 or Lovo cell lines stably expressing Vector or NEK8. Western blots confirmed the successful construction of these cell lines (Fig. 2A). CCK8 assays were carried out to assess the proliferation ability of cancer cells. Results showed that NEK8 overexpression significantly enhanced the proliferation ability of SW48 or Lovo cells (Fig. 2B). Next, we co-cultured EdU and SW48 or Lovo cell lines to validate the function of NEK8 in colorectal cancer cells. The results revealed that NEK8-expressing cells exhibited a higher DNA replication capacity than the Vector control cells (Fig. 2C, D). Additionally, we assessed the ATP levels in both Vector and NEK8 cancer cells. Results showed that the ATP level of NEK8 cancer cells was significantly higher than Vector cancer cells (Fig. 2E). Overall, these results showed that NEK8 overexpression could significantly enhance the proliferation ability of colorectal cancer cells.

### Loss of NEK8 inhibited colorectal cancer cell proliferation in vivo and in vitro

**Fig. 3:**
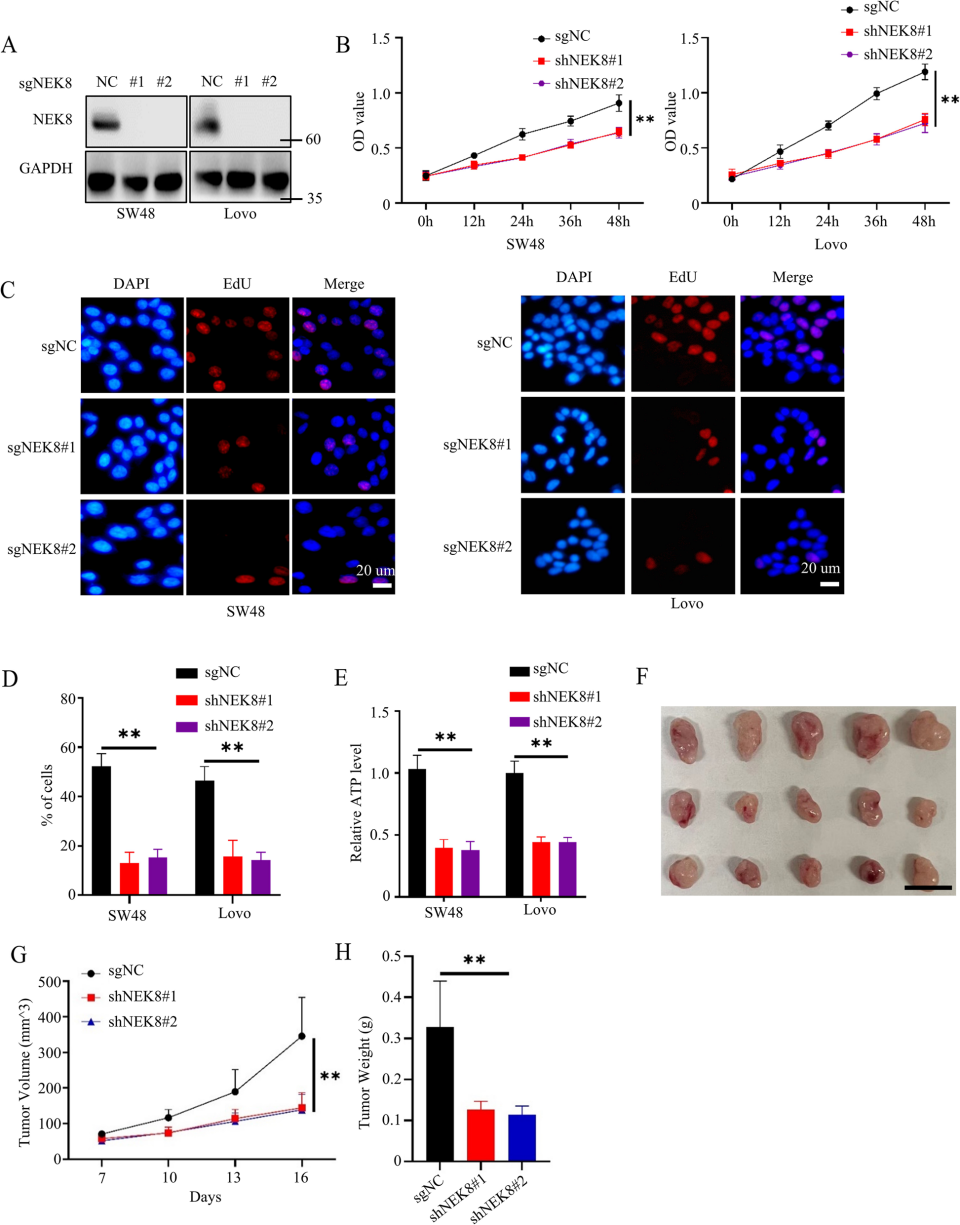
Deletion of NEK8 inhibited colorectal cancer cell proliferation in vivo and in vitro. **A** Knocking out the expression of NEK8 using the Crisp-cas9 system in SW48 and Lovo cell lines; western blots was conducted to confirm successful construction. **B** CCK8 assays to investigate the proliferation ability of SW48 and Lovo from Fig. 3A; *n* = 3, P(Left) = 0.0056 or 0.0065, P(Right) = 0.0012 or 0.0019. **C**, **D** EdU assays to investigate the DNA replication ability of SW48 and Lovo cells from Fig. 3A; representative images shown (**C**); statistical analysis shown (**D**); *n* = 3, P(SW48) = 0.0005, P(Lovo) = 0.0038 or 0.0011. **E **Detecting the cellular ATP level of SW48 and Lovo from Fig. 3A; *n* = 3, P(SW48) = 0.001, P(Lovo) = 0.0008 or 0.0007. **F **The representative tumor images are shown. **G **The tumor growth curve is shown; *n* = 5, *P* = 0.0048 or 0.0042. **H **Statistical analysis of tumor weight; *n* = 5, *P* = 0.0041 or 0.0029. All western blots were conducted three times, and similar results were found; a Student's t-test was applied for statistical analysis

To further validate the pro-proliferation ability of NEK8 in colorectal cancer cells, we knocked out NEK8 expression using the Crispr-cas9 system in SW48 or Lovo cancer cells. Western blots confirmed the successful construction of NEK8 knockout cell lines (Fig. 3A). We substantiated that loss of NEK8 could significantly inhibit the proliferation ability of colorectal cancer cells in vitro by CCK8 assays (Fig. 3B). Moreover, we found that NKE8 knockout significantly decreased the DNA replication ability and the cellular ATP level of cancer cells (Fig. 3C-E). These results showed that silencing NEK8 could suppress the proliferation ability of cancer cells in vitro.

Next, we conducted animal experiments to assess the biological function of NEK8 in colorectal cancer in vivo. We first subcutaneously injected sgNC, sgNEK8#1, or sgNEK8#2 cancer cells into the back of 8-week-old nude mice and measured the tumor volume at the indicated time. Results showed that sgNEK8 tumors grew slower than sgNC tumors, and the weight of sgNEK8 tumors was much lower than that of sgNC tumors (Fig. 3F-H). Besides, we detected the ki-67 level (an indicator of the proliferation ability of cancer cells) of tumors using IHC and western blots. Results showed that loss of NEK8 could significantly inhibit the proliferation ability of cancer cells in vivo (Figure S2A-B). Taken together, these data revealed that NEK8 could positively regulate the proliferation ability of colorectal cancer cells in vivo or in vitro.

### NKE8 positively regulated the MYC signaling pathway

**Fig. 4:**
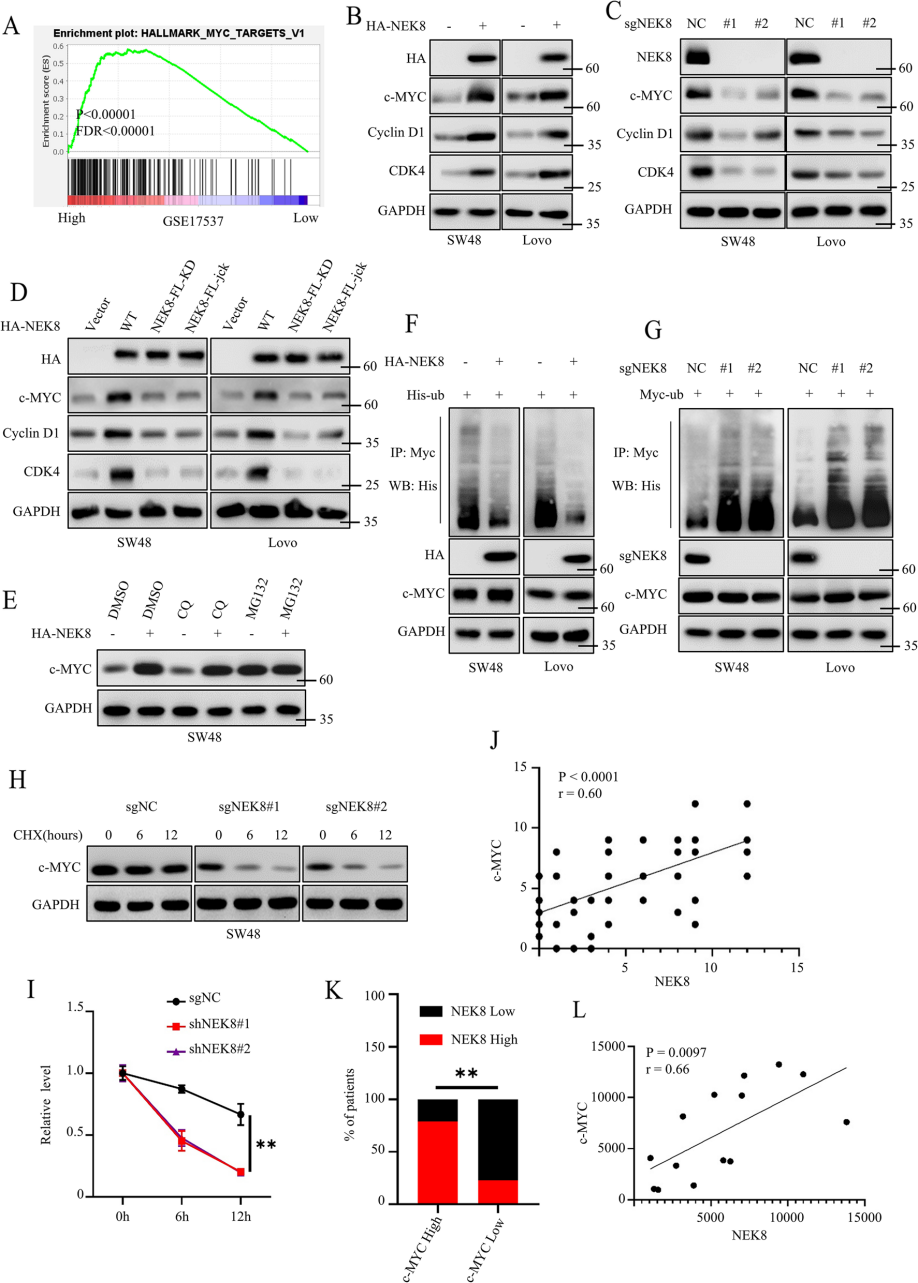
NEK8 positively activated the MYC signaling pathway by regulating the poly-ubiquitin level of c-MYC. **A **GSEA analysis of GSE17537 dataset; *P* < 0.0001, FDR < 0.0001. **B **Western blots to detect the expression level of identified proteins of SW48 and Lovo stably expressing Vector or NEK8. **C **Western blots to detect the expression level of identified proteins of SW48 and Lovo stably expressing sgNC or sgNEK8. **D **Transfecting HA-NEK8 WT and HA-NEK8 enzyme deficient mutants (NEK8-FL-KD and NEK8-FL-jck) into SW48 and Lovo cells; Western blots to detect the expression level of identified protein. **E **Western blots to detect the expression level of identified protein of SW48 treated by DMSO, CQ (20 uM), or MG132 (5 uM). **F **Co-transfecting his-ub plasmid and Vector or HA-NEK8 plasmid into SW48 and Lovo cells; MG132 to inhibit endogenous c-MYC degradation; co-IP and western blot to investigate the poly-ubiquitin level of c-MYC. **G **Transfecting his-ub plasmid into sgNC or sgNEK8 cancer cells; MG132 to inhibit endogenous c-MYC degradation; co-IP and western blot to investigate the poly-ubiquitin level of c-MYC. **H **Cycloheximide (CHX, 50 uM) was applied to SW48 cells stably expressing sgNC or sgNEK8 at the indicated time; western blots to detect the degradation rate of c-MYC. **I **Statistical analysis for Fig. 4H; *n* = 3, *P* = 0.0007 or = 0.0009. **J **Statistical analysis for Figure S3D; Tested by Pearson's correlation test, *n* = 69, *P* < 0.0001. K. IHC score greater than 6 was considered high; otherwise, it was considered low; Statistical analysis for Figure S3D; Tested by Chi-Squared Test, *P* < 0.0001. **L **Statistical analysis for Figure S3E; Tested by Pearson's correlation test, *n* = 14, *P* = 0.0097. All western blots were conducted three times, and similar results were found

Next, we further investigated the detailed mechanism of NEK8-mediated tumor proliferation. We first conducted GSEA analysis using multiple colorectal cancer datasets, including GSE17537, GSE24551, and GSE29623. Results showed that NEK8 could activate MYC signaling pathways (Fig. 4A and Figure S3A). Next, we detected the expression level of c-MYC and its downstream genes (cyclin D1 and CDK4) in SW48 and Lovo ectopically expressing Vector or NEK8 using western blots. We found that NEK8 overexpression could significantly upregulate the expression level of c-MYC, cyclin D1, and CDK4 (Fig. 4B). Consistently, loss of NEK8 significantly inhibited the expression level of c-MYC, cyclin D1, and CDK4 (Fig. 4C). Moreover, we transfected HA-NEK8 WT and HA-NEK8 enzyme-deficient mutants (NEK8-FL-KD and NEK8-FL-jck) into SW48 and Lovo cells. Western blot analysis demonstrated that NEK8-mediated c-MYC, cyclin D1, and CDK4 upregulation were dependent on NEK8 enzyme activation (Fig. 4D). Then, we further investigated how NEK8 regulated c-MYC expression. We first detected the mRNA expression level of c-MYC in Vector, NEK8, sgNC, or sgNEK8 cancer cells. The results showed that the overexpression or silencing of NKE8 did not affect the mRNA expression level of c-MYC (Figure S3B, C), which suggested that NEK8 might regulate c-MYC expression via post-translational mechanisms. It has been reported that the ubiquitin–proteasome and autophagy-lysosome pathways are the main ways for protein degradation. Hence, we used chloroquine (CQ) to inhibit the autophagy-lysosome pathway and MG132 to inhibit the ubiquitin–proteasome pathway. Notably, MG132 application, but not CQ, could significantly upregulate the expression level of c-MYC, which implied that the ubiquitin–proteasome pathway was the main pathway for c-MYC degradation. Besides, MG132 abrogated the NEK8-mediated c-MYC upregulation (Fig. 4E). Then, we investigated whether NEK8 could regulate the poly-ubiquitin level of c-MYC. To inhibit the degradation of endogenous c-MYC, we treated the cells with MG132. Results showed that NEK8 overexpression decreased the poly-ubiquitin level of c-MYC, and loss of NEK8 increased the poly-ubiquitin level of c-MYC (Fig. 4F, G). Besides, c-MYC from sgNEK8 cells exhibited a higher degradation rate than c-MYC from sgNC cells (Fig. 4H, I).

To further confirm the correlation between NEK8 and c-MYC, we analyzed the expression level of NEK8 and c-MYC in 69 cases of colorectal cancer tissues. Representative images are shown in Figure S3D. Statistical analysis showed a positive correlation between the expression levels of NEK8 and c-MYC (Fig. 4J, K). Consistent findings were observed during western blot analysis (Fig. 4L and Figure S3E). RT-PCR revealed no relationship between the mRNA expression levels of NEK8 and c-MYC (Figure S3F). Collectively, these data revealed that NEK8 could positively regulate the MYC signaling pathway by inhibiting the poly-ubiquitin level of c-MYC.

### NEK8 phosphorylated c-MYC at serine 405

**Fig. 5:**
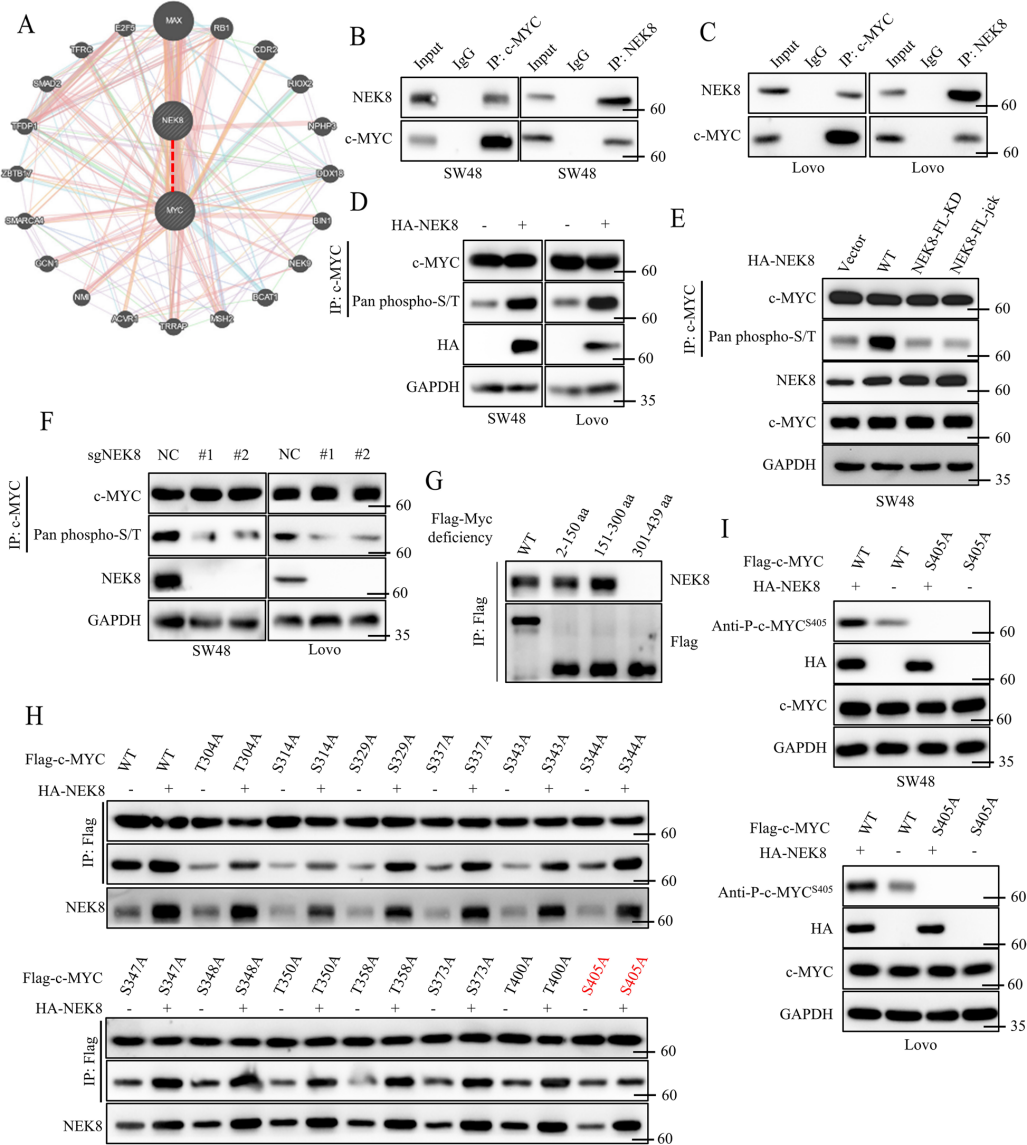
NEK8 phosphorylated c-MYC at serine 405. **A** Analysis of the potential binding of NEK8 and c-MYC using the GENEMANIA database. **B**, **C** Co-IP assays to investigate the potential interaction of NEK8 and c-MYC in SW48 (**B**) or Lovo (**C**) cells. **D **MG132 to inhibit endogenous c-MYC degradation; co-IP and western blot to investigate the poly-ubiquitin level of c-MYC in SW48 and Lovo stably expressing Vector or NEK8. **E** MG132 to inhibit endogenous c-MYC degradation; co-IP and western blot to investigate the poly-ubiquitin level of c-MYC in SW48 and Lovo expressing Vector, NEK8 WT, or NEK8 enzyme deficient mutants (NEK8-FL-KD and NEK8-FL-jck). **F **MG132 to inhibit endogenous c-MYC degradation; co-IP and western blot to investigate the poly-ubiquitin level of c-MYC in SW48 and Lovo stably expressing sgNC or sgNEK8. **G** Separately transfecting Flag-c-MYC segmented plasmid into HEK293T cells; co-IP and western blot to investigate the potential binding between c-MYC and NEK8. **H** Co-transfecting Vector or HA-NEK8 and Flag-c-MYC mutants into HEK293T cells; co-IP and western blot to investigate the phosphorylation level of c-MYC. **I** Transfecting Vector or HA-NEK8 into cancer cells stably expression c-MYC WT or c-MYC S405A, MG132 (5 uM) to inhibit endogenous c-MYC degradation; Anti-P–c-MYC^S405^ antibody to detect the S405 phosphorylation level of c-MYC. All western blots were conducted three times, and similar results were found

Although we established that NEK8 could regulate the poly-ubiquitin level of c-MYC, the exact mechanism remains unclear. Accordingly, we further investigated how NEK8 regulated the poly-ubiquitin level of c-MYC. First, we analyzed the potential binding interaction between NEK8 and c-MYC using the GENEMANIA database. Interestingly, the analysis revealed a possible binding association between NEK8 and c-MYC (Fig. 5A). Then, we co-transfected HA-NEK8 and flag-c-MYC into HEK293T cells, and co-IP analysis showed that NEK8 could bind to c-MYC (Figure S4A). Endogenous co-IP assays confirmed the interaction between NEK8 and c-MYC (Fig. 5B-C). As previously reported, NKE8 functions as a serine/threonine protein kinase. Our study provided compelling evidence that NEK8 enzyme activity is essential for upregulating NEK8-mediated c-MYC expression (Fig. 4D). Hence, we wondered whether NEK8 could phosphorylate c-MYC. We first co-transfected HA-NEK8 and Flag-c-MYC plasmids into HEK293T cells, and a pan-serine/threonine phosphorylation antibody was applied to detect the phosphorylation level of c-MYC. Results showed that NEK8 overexpression could significantly upregulate the phosphorylation level of c-MYC (Figure S4B). We next transfected HA-NEK8 WT, HA- NEK8-FL-KD, or NEK8-FL-jck mutant into SW48 and Lovo cells, and MG132 was used to inhibit the degradation of endogenous c-MYC. Results confirmed that NEK8 WT, but not NEK8-FL-KD or NEK8-FL-jck, overexpression could regulate the serine/threonine phosphorylation of c-MYC (Fig. 5D-E). Similarly, silencing NEK8 could significantly inhibit the serine/threonine phosphorylation of c-MYC (Fig. 5F). We next conducted experiments to identify the specific serine/threonine residues that NEK8 could phosphorylate. First, we constructed Flag-c-MYC segmented plasmid (deleting amino acid from 2nd to 150th, 151st to 300th, or 301st to 439th). We transfected Flag-c-MYC WT or Flag-c-MYC segmented plasmids into HEK293T cells, followed by co-IP assays. The results demonstrated that the deletion of amino acids from the 301st to 439th region of c-MYC abolished the interaction between c-MYC and NEK8, as shown in Fig. 5G. This suggests that the phosphorylation site catalyzed by NEK8 may reside within the 301st to 439th amino acid region of c-MYC. Next, we separately mutated every serine (S) or threonine (T) to alanine (A), and 13 mutant plasmids were constructed successfully. We co-transfected HA-NEK8 and Flag-c-MYC WT or mutants into HEK293T cells. Results showed that the c-MYC S405A mutant abrogated NEK8-mediated c-MYC phosphorylation upregulation (Fig. 5H), suggesting that NEK8 might catalyze the serine 405 phosphorylation of c-MYC. To validate this hypothesis, we generated a specific antibody that can recognize the phosphorylation of serine 405 in c-MYC (Anti-P–c-MYC^S405^). We first co-transfected HA-NEK8 and Flag-c-MYC WT or Flag-c-MYC S405A mutant into HEK293T cells and used anti-P–c-MYC^S405^ to detect the phosphorylation level of c-MYC. Results showed that the anti-P–c-MYC^S405^ antibody did not detect the phosphorylation signaling in c-MYC S405A mutant protein, which showed that anti-P–c-MYC^S405^ antibody could specifically recognize the S405 phosphorylation of c-MYC, and the c-MYC S405A mutant completely abrogated NEK8-mediated c-MYC phosphorylation upregulation (Figure S4C). Furthermore, we knocked out c-MYC expression in SW48 and Lovo cells using the Crispr-cas9 system (Figure S4D). Meanwhile, we constructed SW48 and Lovo cell lines stably expressing c-MYC WT or the c-MYC S405A mutant (Figure S4E). We transfected HA-NEK8 plasmids into SW48 or Lovo cells stably expressing c-MYC WT or c-MYC S405A mutant. Analysis of western blots confirmed that the c-MYC S405A mutant completely abrogated NEK8-mediated c-MYC phosphorylation (Fig. 5I). Besides, we substantiated that NEK8 enzyme activation was necessary for NEK8-mediated c-MYC phosphorylation upregulation (Figure S4F). Taken together, these results showed that NEK8 could phosphorylate c-MYC at serine 405.

### S405 phosphorylation stabilized c-MYC by blocking its ploy-ubiquitin and promoted colorectal cancer cell proliferation in vitro

**Fig. 6:**
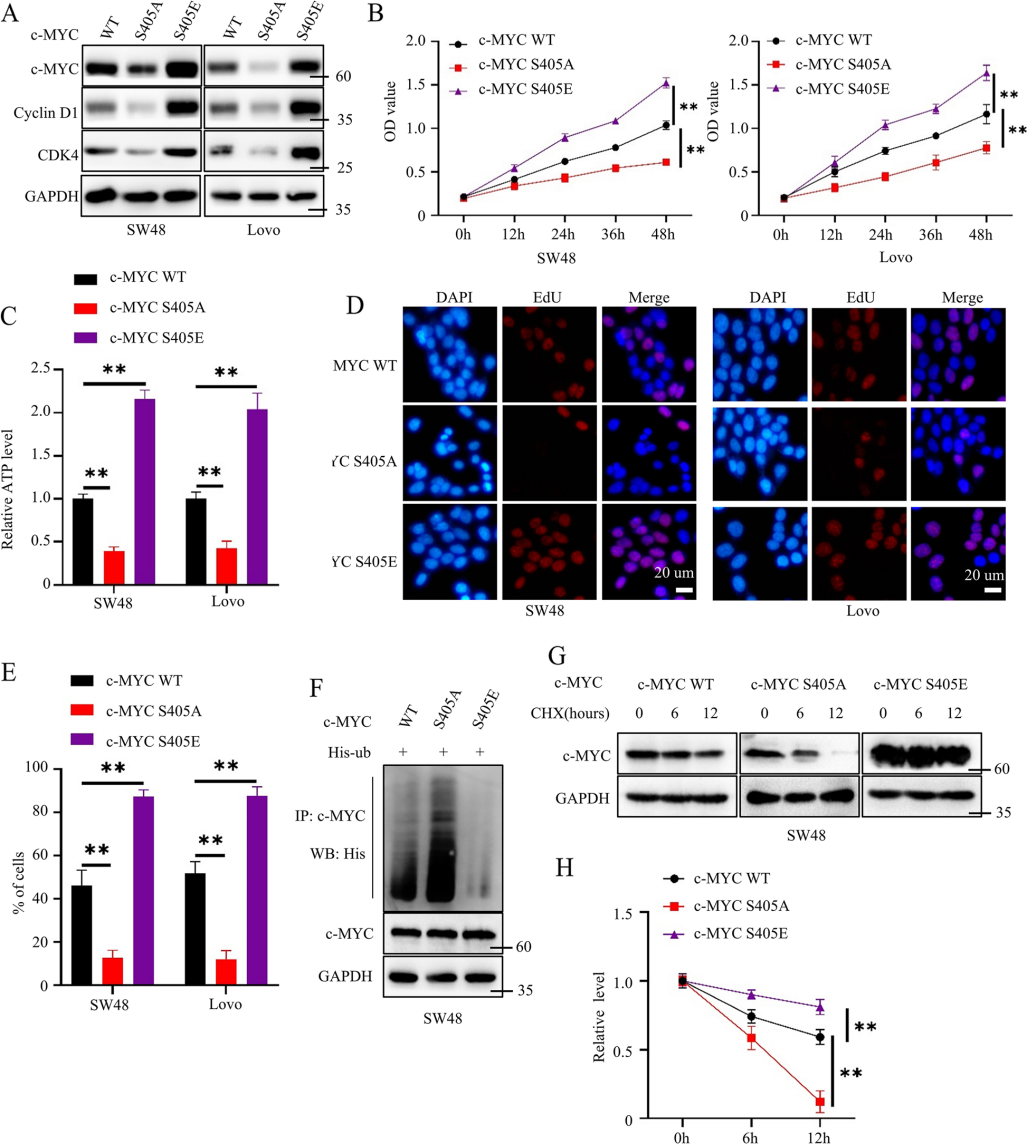
S405 phosphorylation of c-MYC blocked its poly-ubiquitin. **A** Western blots to detect the expression level of c-MYC, cyclin D1, and CDK4 in cancer cells stably expressing c-MYC WT, c-MYC S405A, or c-MYC S405E. **B** CCK8 assays to detect the proliferation ability of cancer cells stably expressing c-MYC WT, c-MYC S405A, or c-MYC S405E; *n* = 3, P(Left) = 0.0004 or 0.0002, P(Right) = 0.0071 or 0.0044. **C** Detecting the cellular ATP level of cancer cells stably expressing c-MYC WT, c-MYC S405A, or c-MYC S405E; *n* = 3, P(SW48) = 0.0001 or < 0.0001, P(Lovo) = 0.0009. **D**, **E** EdU assays to investigate the DNA replication ability of cancer cells stably expressing c-MYC WT, c-MYC S405A, or c-MYC S405E; representative images shown (**D**); statistical analysis shown (**E**); *n* = 3, P(SW48) = 0.002 or = 0.0008, P(Lovo) = 0.0005 or = 0.0008. **F** MG132 to inhibit c-MYC degradation; co-IP and western blots to detect the poly-ubiquitin level of c-MYC in SW48 cells stably expressing c-MYC WT, c-MYC S405A, or c-MYC S405E. **G** Cycloheximide (CHX, 50 uM) was applied to SW48 cells stably expressing c-MYC WT, c-MYC S405A, or c-MYC S405E at the indicated time; western blots to detect the degradation rate of c-MYC. **H** Statistical analysis for Fig. 6G; *n* = 3, *P* = 0.001 or = 0.008. All western blots were conducted three times, and similar results were found; a Student's t-test was applied for statistical analysis

To further investigate the function of the S405 phosphorylation of c-MYC, we first constructed SW48 and Lovo cells stably expressing c-MYC S405A mutant (a phosphorylation deficiency mutant) and c-MYC S405E (a phosphorylation mimetic mutant). MG132 was applied to inhibit the c-MYC degradation. Western blot results showed that c-MYC expression was comparable in c-MYC WT, c-MYC S405A, and c-MYC S405E cancer cells (Figure S4G). However, we found that c-MYC S405A cancer cells had lower c-MYC, cyclin D1, and CDK4 expression than c-MYC WT cancer cells, while c-MYC S405E cancer cells had higher c-MYC, cyclin D1, and CDK4 expression than c-MYC WT cancer cells (Fig. 6A). Meanwhile, we found that c-MYC S405A cancer cells had lower proliferation ability, cellular ATP level, and DNA replication ability than c-MYC WT cancer cells, while c-MYC S405E cancer cells had higher proliferation ability, cellular ATP level, and DNA replication ability than c-MYC WT cancer cells (Fig. 6B-E). Moreover, we detected the poly-ubiquitin level of c-MYC WT, c-MYC S405A, and c-MYC S405E. MG132 was applied to inhibit c-MYC degradation. Results showed that the c-MYC S405A protein exhibited a higher poly-ubiquitin level than the c-MYC WT protein, while the c-MYC S405E protein had a lower poly-ubiquitin level than the c-MYC WT protein (Fig. 6F). Besides, c-MYC S405A protein exhibited a higher degradation rate than c-MYC WT protein, while c-MYC S405E protein displayed a lower degradation rate than c-MYC WT protein (Fig. 6G, H). Collectively, we found that the c-MYC S405 phosphorylation stabilized the c-MYC protein and promoted colorectal cancer cell proliferation in vitro.

### NEK8-mediated colorectal tumor proliferation dependent on MYC signaling in vivo and in vitro

**Fig. 7:**
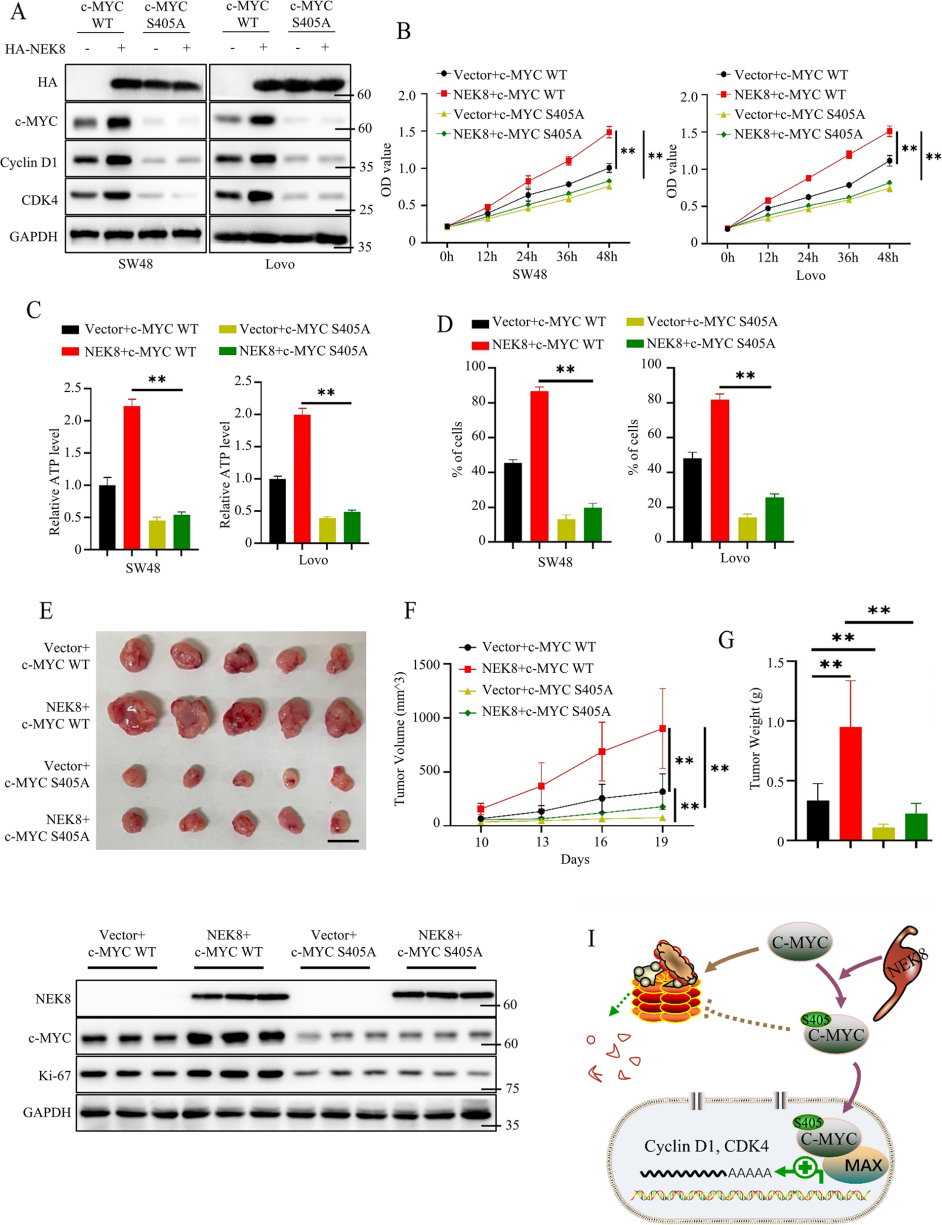
NEK8-mediated colorectal cancer cell proliferation depended on c-MYC S405 phosphorylation. **A** Western blots to detect the expression level of c-MYC, cyclin D1, and CDK4 in cancer cells stably expressing Vector + c-MYC WT, NEK8 + c-MYC WT, Vector + c-MYC S405A, NEK8 + c-MYC S405A. **B** CCK8 assays to detect the proliferation ability of cancer cells from Fig. 7A; *n* = 3, P(SW48) = 0.0011 or 0.0001, P(Lovo) = 0.0025 or 0.0001. **C** Detecting the cellular ATP level of cancer cells from Fig. 7A; *n* = 3, P(SW48) < 0.0001, P(Lovo) < 0.0001. **D **EdU assays to investigate the DNA replication ability of cancer cells from Fig. 7A; statistical analysis shown; *n* = 3, P(SW48) < 0.0001, P(Lovo) < 0.0001. **E** The representative tumor images are shown. **F** The tumor growth curve is shown; *n* = 5, *P* = 0.0103, 0.0072, or 0.0035. **G** Statistical analysis of tumor weight; *n* = 5, *P* = 0.0121, 0.0113, or 0.0024. **H** Western blots to investigate the expression level of NEK8, c-MYC, and Ki-67 in tumors from Fig. 7E. **I **The working model is shown. All western blots were conducted three times, and similar results were found; a Student's t-test was applied for statistical analysis

To further clarify the correlation between NEK8 signaling and MYC signaling in colorectal cancer progress, we constructed SW48 and Lovo cells stably expressing Vector + shNC, NEK8 + shNC, NEK8 + shc-MYC#1, NEK8 + shc-MYC#2. We found that the NEK8-mediated increase in cyclin D1 and CDK4 was dependent on c-MYC (Figure S5A). Besides, we found that silencing of c-MYC could abrogate NEK8-mediated increase in cancer cell proliferation, cellular ATP, and DNA replication (Figure S5B-E). These findings revealed that MYC signaling was essential for NEK8-mediated colorectal tumor proliferation. However, the role of c-MYC S405 phosphorylation in NEK8/c-MYC signaling remained unclear. Next, we constructed SW48 and Lovo cells stably expressing Vector + c-MYC WT, NEK8 + c-MYC WT, Vector + c-MYC S405A, NEK8 + c-MYC S405A. Results from western blots showed that overexpression of NEK8 could upregulate c-MYC, cyclin D1, and CDK4 expression in c-MYC WT cells but not in c-MYC S405A cells (Fig. 7A). CCK8 assays also proved that NEK8 overexpression could promote the proliferation ability of c-MYC WT cells, while c-MYC S405A abrogated this effect (Fig. 7B). Moreover, NEK8 overexpression could increase the cellular ATP level and the DNA replication ability in c-MYC WT cells but not in c-MYC S405A cells (Fig. 7C, D and Figure S5F). Next, we further assessed the relationship between NEK8 signaling and MYC signaling in vivo. We subcutaneously injected these four kinds of cancer cells (SW48 stably expressing Vector + c-MYC WT, NEK8 + c-MYC WT, Vector + c-MYC S405A, NEK8 + c-MYC S405A) into the back of 8-week-old nude mice. Tumor volume was measured at the indicated time. Results showed that the growth rate and weight of tumors induced by c-MYC S405A cells were significantly lower than tumors induced by c-MYC WT cells, and the c-MYC S405A mutant abrogated the NEK8-mediated increase in tumor proliferation ability (Fig. 7E-G). Meanwhile, we detected the Ki-67 expression of tumors using western blots. Results showed that the expression level of Ki-67 in tumors induced by c-MYC S405A cells was significantly lower than in tumors induced by c-MYC WT cells, and NEK8 overexpression induced the Ki-67 expression of tumors induced by c-MYC WT cells, which was not observed in tumors induced by c-MYC S405A cells (Fig. 7H). Collectively, these findings suggest that c-MYC S405 phosphorylation is essential for NEK8-mediated colorectal tumor progression.

## Discussion

Targeting MYC signaling has long been considered an effective therapeutic strategy for cancer [16, 28]. However, due to the lack of a hydrophobic pocket, groove, and catalytic activity, MYC has been challenging to target with small molecules. Besides, MYC is reportedly located in the cytoplasm and nucleus, making it inaccessible for monoclonal antibodies [16]. Current approaches for targeting MYC mainly include blocking MYC-MAX binding, inhibiting MYC gene transcription, suppressing MYC mRNA translation, destabilizing MYC mRNA, and destabilizing MYC protein. An increasing body of evidence suggests that post-translational modifications of proteins could regulate protein–protein binding, subcellular localization, stabilization, and enzyme activity [16, 29, 30]. Accordingly, revealing novel post-translational modification sites of MYC might provide an effective therapeutic target for colorectal cancer patients. In our study, we revealed that the serine 405 phosphorylation of c-MYC was important for stabilizing c-MYC protein by attenuating the poly-ubiquitin of c-MYC protein. However, it was still unclear how c-MYC S405 phosphorylation regulate ubiquitination of c-MYC in our study. It might be that c-MYC S405 phosphorylation affect the binding between c-MYC and the E3 ubiquitin ligases or deubiqitinating enzymes, while the deeper researches should be conducted to verify the assume. We also proved that loss of serine 405 phosphorylation significantly inhibited the proliferation ability of colorectal cancer cells in vivo and in vitro, and simulated serine 405 phosphorylation significantly enhanced the proliferation ability of colorectal cancer cells in vitro. These results showed that the serine 405 phosphorylation of c-MYC might be a target for colorectal cancer treatment.

Although there have been several studies about the biological function of NEK8, most were about non-neoplastic disease, and the role of NEK8 in tumorigenesis and tumor progression remains unclear. Earlier studies have demonstrated that NEK8 exhibits heightened expression levels in breast cancer, contributing to its overexpression. Furthermore, NEK8 has been found to facilitate the proliferation of gastric cancer cells. Additionally, there is evidence suggesting that NEK8 might influence the infiltration of immune cells in glioma [8, 12, 13]. However, the detailed mechanism of NEK8-mediated tumor proliferation has been largely understudied. In our research, we innovatively revealed that NEK8 was significantly upregulated in colorectal cancer and positively regulated colorectal cancer cell proliferation. In this respect, NEK8 could post-translationally regulate c-MYC expression via phosphorylating c-MYC at the serine 405 sites. Besides, we revealed a positive correlation between the expression levels of NEK8 and c-MYC in colorectal tissues. However, a study showed that NEK8 R599X nonsense mutation induced nonsense-mediated decay (NMD) of NEK8, which could upregulate the mRNA level of c-MYC and cause multiple organ dysplasia and developmental phenotypes. The heterogeneity in findings may be attributed to the different experimental conditions, cell lines, and diseases. We also demonstrated that NEK8-induced colorectal cancer proliferation relied on the serine 405 phosphorylation of c-MYC. Meanwhile, the deletion of NKE8 significantly attenuated the proliferation ability of colorectal cancer cells. Overall, these findings provided the theoretical basis for pharmacologically inhibiting the enzymatic activity of NEK8 for colorectal cancer treatment.

In conclusion, we innovatively revealed the biological function of NEK8 in colorectal cancer. We corroborated that NEK8-mediated colorectal cancer cell proliferation depends on the phosphorylation of c-MYC at the serine 405 sites. Based on these findings, we propose that the NEK8/c-MYC signaling pathway could be a potential therapeutic target for colorectal cancer.

### Supplementary Information

**Additional file 1.**

## Data Availability

Not applicable.
