# Supplementary material for: NEK8 regulates colorectal cancer progression via phosphorylating MYC
Source: Cell Commun Signal. 2023 Aug 18;21:209. doi: 10.1186/s12964-023-01215-z (PMC10436496; doi:10.1186/s12964-023-01215-z)

**Supplementary figure legend**

**Supplementary Figure 1. NEK8 was overexpressed in multiple kinds of tumors. A**. Analysis of the expression level of NEK8 in multiple kinds of tumor tissues and normal tissues using the GEPIA database. **B**. Kaplan-Meier Plotter of multiple kinds of tumor patients grouped by the expression level of NEK8.


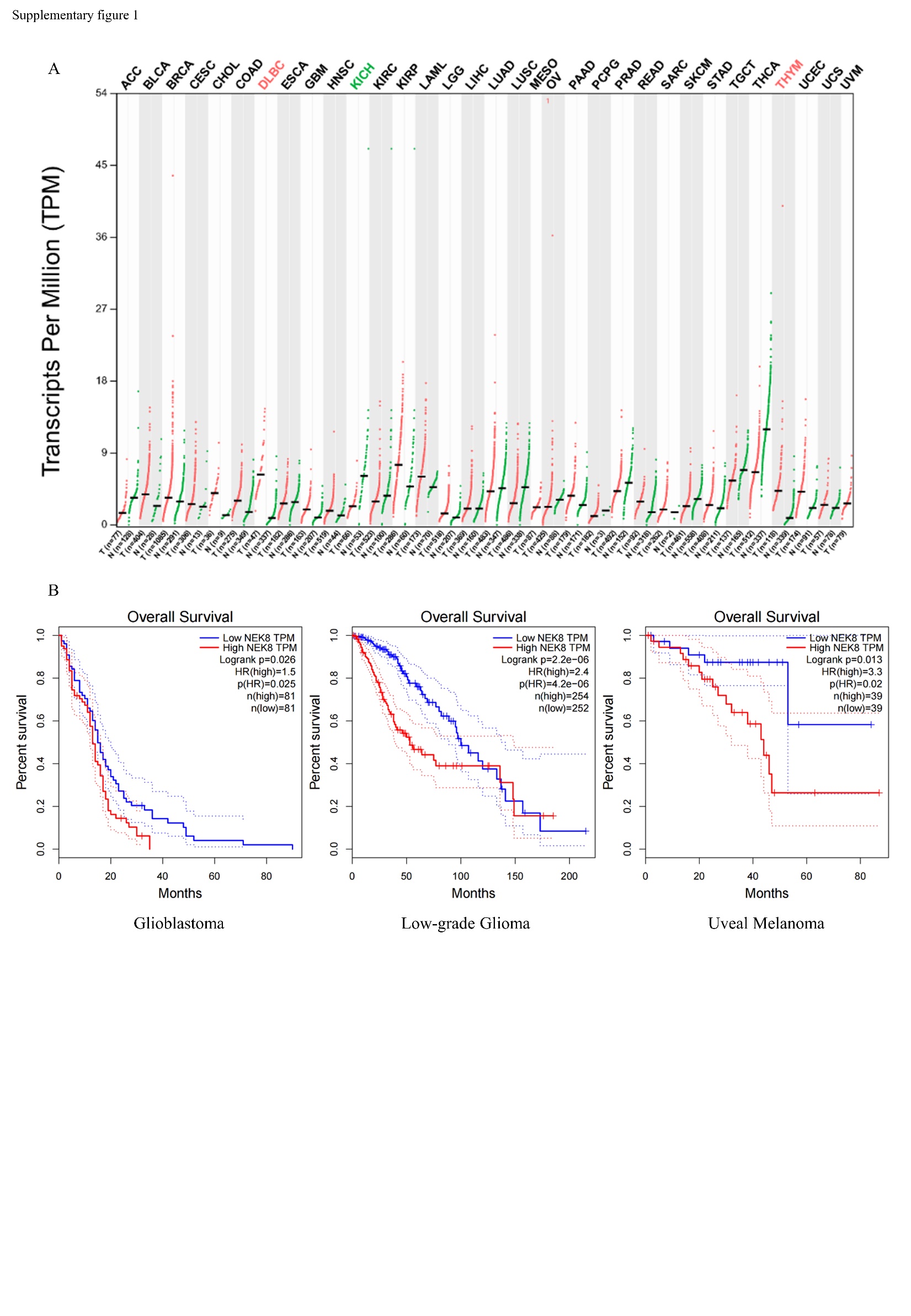


**Supplementary Figure 2. Loss of NEK8 attenuated the proliferation ability of colorectal cancer cells in vivo. A**. IHC assays to investigate the expression level of NEK8, c-MYC, and Ki-67 in tumors from **Figure 3F**. **B**. Western blots to investigate the expression level of NEK8, c-MYC, and Ki-67 in tumors from **Figure 3F**.


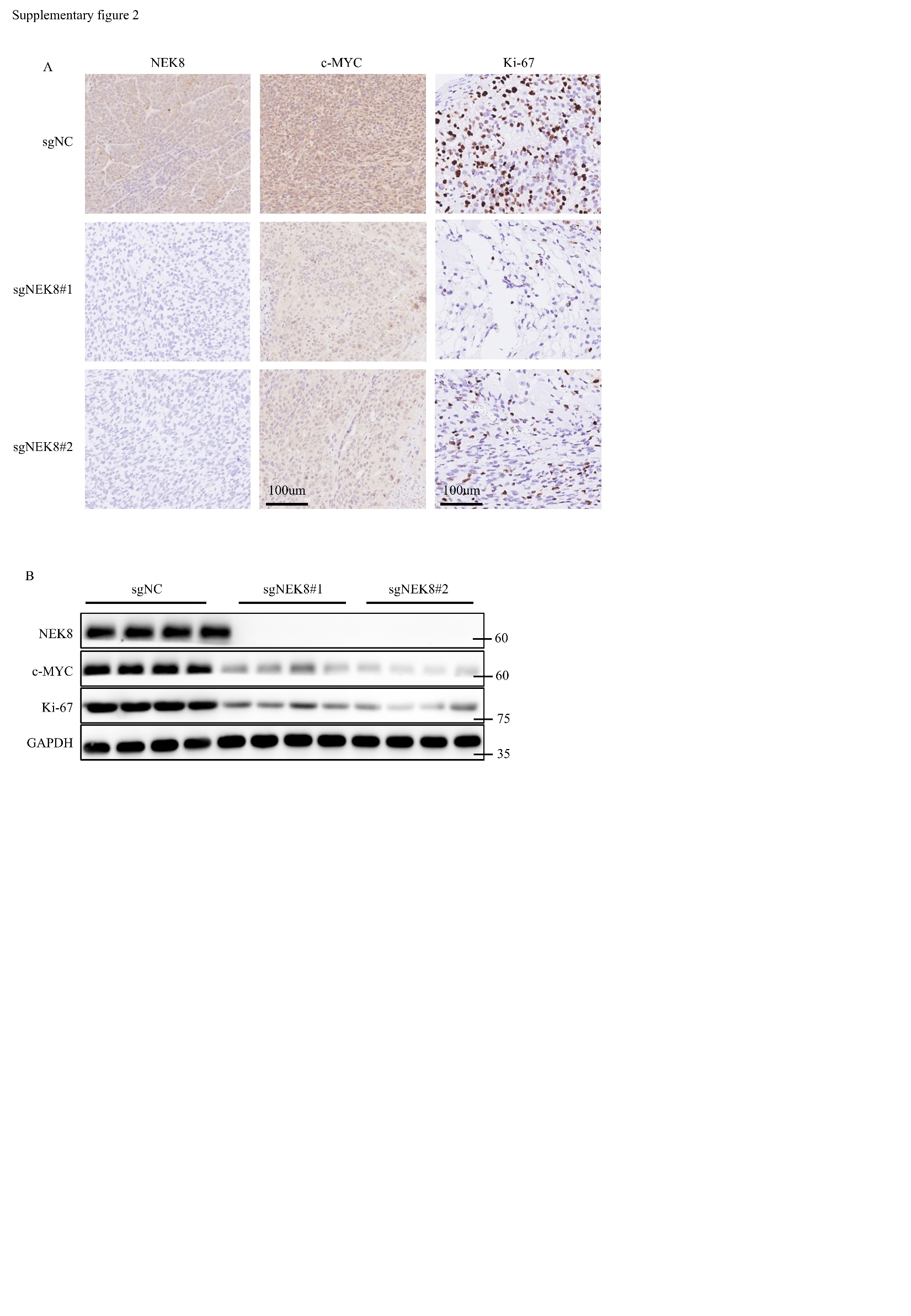


**Supplementary Figure 3. NEK8 positively activated the MYC signaling pathway. A**. GSEA analysis of GSE24551 and GSE29623 dataset; P (GSE24551) = 0.011, FDR (GSE24551) = 0.20, P (GSE29623) = 0.016, FDR (GSE29623) = 0.19. **B**. RT-PCR assays to detect the mRNA expression level of c-MYC in SW48 and Lovo cells stably expressing Vector or NEK8. **C**. RT-PCR assays to detect the mRNA expression level of c-MYC in SW48 and Lovo cells stably expressing sgNC or sgNEK8. **D**. IHC assays to detect the expression level of NEK8 and c-MYC in 69 cases of colorectal tumors; Representative images are shown. **E**. Western blots to detect the expression level of NEK8 and c-MYC in 14 cases of colorectal tumors. **F**. RT-PCR assays to the mRNA expression level of c-MYC and NEK8 in 20 cases of colorectal tumors; Statistical analysis shown, Tested by Pearson's correlation test, P = 0.5548. All western blots were conducted three times, and similar results were found.

**Supplementary Figure 4. The S405 of c-MYC was phosphorylated by NEK8. A**. Co-transfecting HA-NEK8 and Flag-c-MYC plasmids into HEK293T cells; co-IP and western blot to investigate the possible binding of NEK8 and c-MYC. **B**. Co-transfecting Vector or HA-NEK8 and Flag-c-MYC plasmids into HEK293T cells; co-IP and western blot to investigate the phosphorylation level of c-MYC. **C**. Co-transfecting Vector or HA-NEK8 and Flag-c-MYC WT or Flag-c-MYC S405A plasmids into HEK293T cells; co-IP and western blot to investigate the S405 phosphorylation level of c-MYC. **D**. Knocking out c-MYC expression in SW48 and Lovo cells using Criap-cas9 system; western blots to confirm the successful construction. **E**. MG132 to inhibit c-MYC degradation; western blots to detect the expression level of c-MYC in c-MYC WT and c-MYC S405A cells. **F**. MG132 to inhibit endogenous c-MYC degradation; co-IP and western blot to investigate the poly-ubiquitin level of c-MYC in SW48 and Lovo expressing Vector, NEK8 WT, or NEK8 enzyme deficient mutants (NEK8-FL-KD and NEK8-FL-jck). **G**. MG132 to inhibit c-MYC degradation; western blots to detect the expression level of c-MYC in cancer cells stably expressing c-MYC WT, c-MYC S405A, or c-MYC S405E. All western blots were conducted three times, and similar results were found.


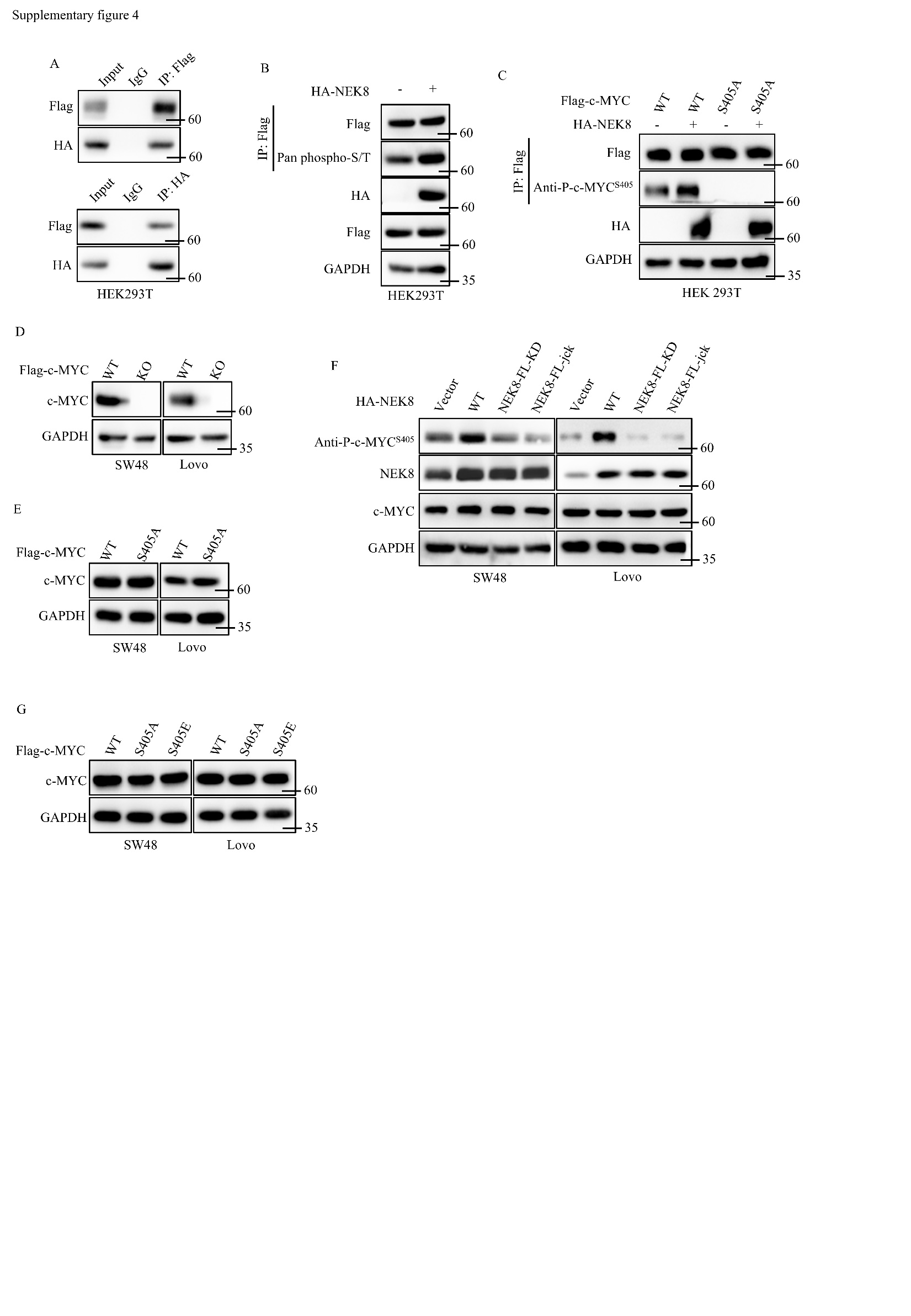


**Supplementary Figure 5. NEK-mediated tumor proliferation relied on c-MYC. A**. Western blots to detect the expression level of c-MYC, cyclin D1, and CDK4 in cancer cells stably expressing Vector+shNC, NEK8+shNC, NEK8+shc-MYC#1, NEK8+shc-MYC#2. **B**. CCK8 assays to detect the proliferation ability of cancer cells from Figure S5A; n = 3, P(SW48) = 0.0006, P(Lovo) = 0.0007 or < 0.0001. **C**-**D**. EdU assays to investigate the DNA replication ability of cancer cells from Figure S5A; representative images shown (**C**), statistical analysis shown (**D**); n=3, P(SW48) < 0.0001 or = 0.0003, P(Lovo) = 0.0002 or 0.0004. **E**. Detecting the cellular ATP level of cancer cells from Figure S5A; n=3, P(SW48) = 0.0004 or 0.0007, P(Lovo) < 0.0001. **F**. EdU assays to investigate the DNA replication ability of cancer cells from **Figure S4A**; representative images are shown. All western blots were conducted three times, and similar results were found; a Student's t-test was applied for statistical analysis.


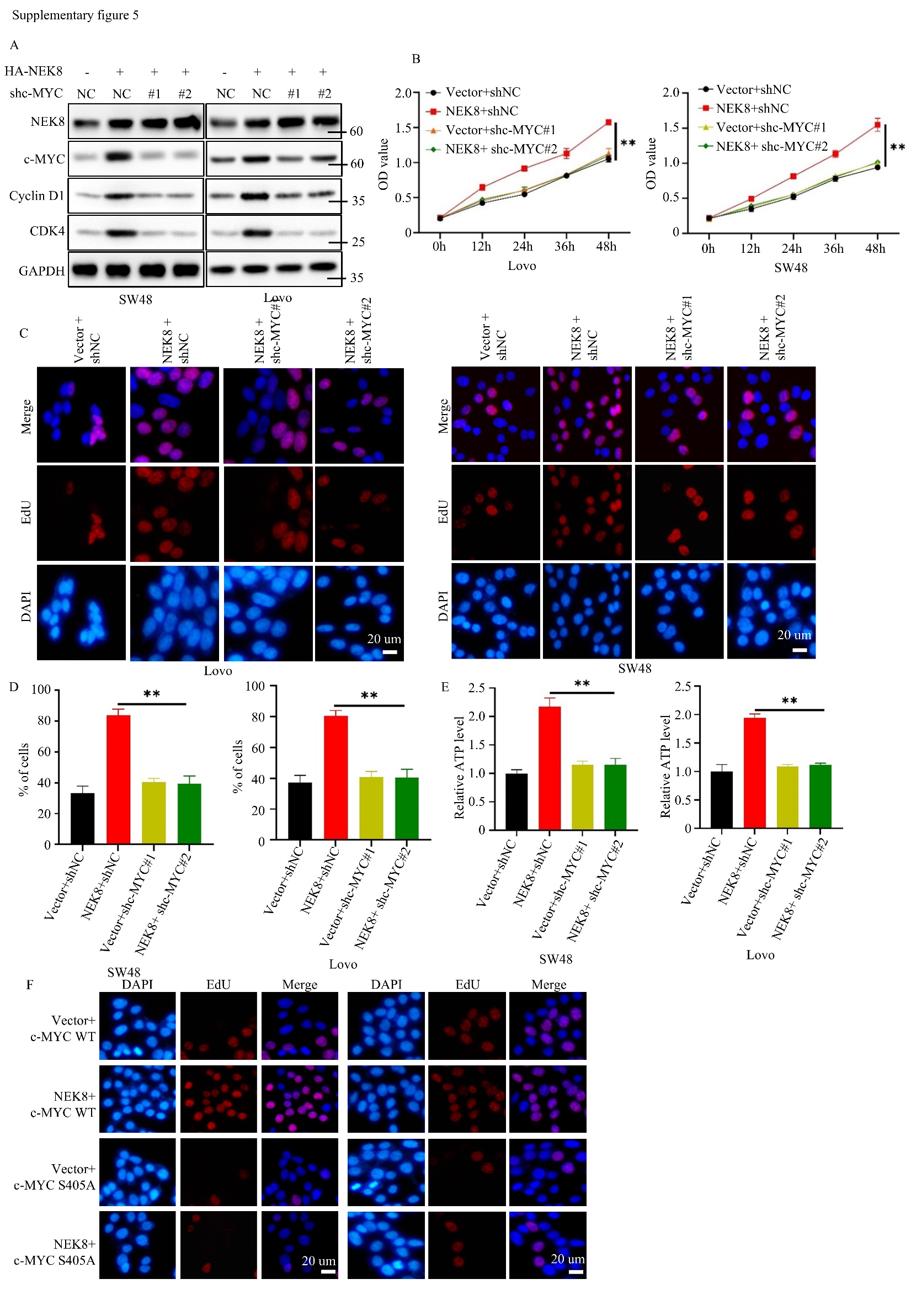

Supplement: Supplementary file 2 — Additional file 1. [file 12964_2023_1215_MOESM1_ESM.docx]
